# Ten-year incidence and impact of coronavirus infections on incidence, etiology, and antimicrobial resistance of healthcare-associated infections in a critical care unit in Western Qatar

**DOI:** 10.5339/qmj.2023.11

**Published:** 2023-07-28

**Authors:** Humberto Guanche Garcell, Jameela Al-Ajmi, Ariadna Villanueva Arias, Joji C Abraham, Angel M Felipe Garmendia, Tania M Fernandez Hernandez

**Affiliations:** ^1^Infection Control Department, The Cuban Hospital. E-mail: humbertoguanchegarcell@yahoo.es ORCID: https://orcid.org/0000-0001-7279-0062; ^2^Corporate Infection Control Department, Hamad Medical Corporation, Qatar

**Keywords:** Healthcare-associated infections, device-associated infections, COVID-19, etiology, antimicrobial resistance, intensive care unit, incidence, Qatar

## Abstract

Background: Healthcare-associated infections (HAI) in critical patients affect the quality and safety of patient care as they impact morbidity and mortality. During the COVID-19 pandemic, an increase in the incidence rate was reported worldwide. We aim to describe the incidence of HAI in the intensive care unit (ICU) during a 10-year follow-up period and compare the incidence during the pre-COVID-19 and COVID-19 periods.

Methods: A retrospective observational study of HAI in the medical-surgical ICU at The Cuban Hospital was conducted. The data collected include the annual incidence of HAI, its etiology, and antimicrobial resistance, using the Centers for Disease Control and Prevention definitions, except for other respiratory tract infections (RTIs).

Results: A total of 155 patients had HAI, of which 130 (85.5%) were identified during COVID-19. The frequency of device-associated infections (DAI) and non-DAI was higher during COVID-19, except for *Clostridium difficile* infections. Etiology was frequently related to species of *Enterobacter*, *Klebsiella*, and *Pseudomonas* in both periods, and a higher frequency of *Acinetobacter*, *Enterococcus*, *Candida*, *Escherichia coli*, *Serratia marcescens*, and *Stenotrophomonas maltophilia* was noted during the COVID-19 period. Device utilization ratio increased by 10.7% for central lines and 12.9% for ventilators, while a reduction of 15% in urinary catheter utilization ratio was observed. DAI incidence was higher during the COVID-19, with a 2.79 higher risk of infection (95% CI: 0.93–11.21; *p* < 0.0050), 15.31 (2.53–625.48), and 3.25 (0.68–31.08) for CLABSI, VAP, and CAUTI, respectively.

Conclusion: The incidence of DAI increased during the pandemic period as compared to the pre-pandemic period, and limited evidence of the impact on antimicrobial resistance was observed. The infection control program should evaluate strategies to minimize the impact of pandemics on HAI.

## Introduction

Healthcare-associated infections (HAI) in critical patients impact the quality and safety of patient care and their morbidity and mortality. Its negative contribution to the efficiency of the healthcare system is in relation to the prolongation of hospital stays, antimicrobial use, and other cost-related issues.^1^

Reducing device-associated infections (DAI) has become a priority for infection prevention in critical patients, and the incidence of central line-associated bloodstream infections (CLABSI), catheter-associated urinary tract infections (CAUTI), and ventilator-associated pneumonia (VAP) constitute key infection control indicators mandated by the Joint Commission International.^2^ DAI prevention and control during the pre-pandemic period had shown progress in reducing its incidence and other adverse outcomes, according to various reports.^3–6^

The COVID-19 pandemic has significantly impacted healthcare systems, including the quality and safety of global healthcare. The infection prevention and control program was seriously affected, so the HAI incidence increased as well as harmed staff safety and the efficiency of healthcare.^6−14^ A recently published report from the Centers for Disease Control (CDC/NHSN) brought evidence about the higher incidence of DAI in 2020 as compared to 2019, emphasizing ventilator-associated events (VAE) and CLABSI.^3^ Moreover, studies published about HAI in various settings describe similar findings, mainly related to the convergence of factors generating a high-risk environment for acquiring infections.^6–16^

The Cuban Hospital (Dukhan, Qatar), between 2020 and 2021, was dedicated to providing care to COVID-19 patients. Originally a 75-bed public hospital, the Joint Commission International-accredited Hamad Medical Corporation (HMC), which is Qatar’s primary healthcare provider, grew to 385 beds, including tents.^17^ The evidence from surveillance suggests an increase in the incidence of HAI as compared to the pre-pandemic period, mostly in critically ill patients. The facility’s infection control program is guided by the corporate infection control program.

Considering the evidence, we conducted a study aimed at describing the incidence of HAI in the ICU during a 10-year follow-up period and comparing the incidence during the pre-COVID-19 and COVID-19 periods.

## Methods

A retrospective observational study of HAI in the medical-surgical intensive care unit (ICU) at The Cuban Hospital was conducted. During the pre-COVID-19 period (2012–2019), the ICU had a 6-bed capacity, and during the COVID-19 pandemic (2020–2021), the capacity was raised to 85 beds.

Data were collected from the Infection Control Department records. The surveillance system was conducted by an infection control practitioner and a hospital epidemiologist. Due to potential variations in the infection control staff during the period 2017–2019, data were validated through a review of patient’s medical records and reports from the microbiology laboratory. The collected data included the annual incidence of HAI, etiology, and antimicrobial resistance. Data based on etiology and antimicrobial resistance were verified using the patient’s electronic medical record. HAI was confirmed using the Centers for Disease Control and Prevention (USA) definitions as per the corporate infection control program, except for other respiratory tract infections (RTIs).^18^ The RTI was confirmed when a positive culture from the respiratory tract (sputum, tracheal aspirate, and other respiratory samples) showed evidence of sepsis (clinical symptoms or sepsis laboratory markers), and VAE were ruled out. DAI, such as CLABSI, CAUTI, and VAP, as well as non-device-associated infections (skin infections (SKIN), decubitus ulcer infections (DECU), and symptomatic urinary tract infections (SUTI)), were reported. Microbiology laboratory results, including etiology and antimicrobial resistance, were considered.

## Analysis

Descriptive statistical methods were used. DAI was presented as rates (1,000 procedures days), percentile distribution, and non-device-associated infection as a percentage. Statistical analysis was done using the statistical package SPSS, version 22 (SPSS Inc., an IBM company, Chicago, IL). Relative risk (RR) ratios, 95% confidence intervals (CIs), and *p*-values were determined. We used the student t-test to compare the HAI incidence between pre-COVID-19 and COVID-19 periods. A two-sided α of <0.05 was considered statistically significant.

## Results

During the study period, 155 patients with HAI were reported, of whom 130 (85.5%) were identified during the COVID-19 pandemic period. The frequency of DAI and non-device-associated infections was higher during the COVID-19 pandemic period, except for *Clostridium difficile* infection. The most frequently reported infection was other RTIs, not classified as VAP. In patients with coronavirus infection, there were two confirmed cases of bloodstream infection (BSI) related to peripheral venous lines and four cases of DECU, SKIN associated with tracheostomy (5 patients), and SUTI 1b (1 case) (Table 1).

The etiology of infections was frequently related to the species of *Enterobacter*, *Klebsiella*, and *Pseudomonas* in both periods, with a higher frequency of *Acinetobacter*, *Enterococcus*, *Candida*, *Escherichia coli*, *Serratia marcescens*, and *Stenotrophomonas maltophilia* during the COVID-19 pandemic period (Table 2).

Table 3 describes the DAI incidence and utilization ratio during the 10-year study period. The incidence was 0.8, 2.6, and 2.9 per 1,000 urinary catheters, central lines, and ventilators for CAUTI, CLABSI, and VAP, respectively. The device utilization ratio was 0.49 for urinary catheters, 0.32 for central lines, and 0.31 for ventilators.

In the pre-COVID-19 (2012–2019) and COVID-19 (2020–2021) periods, 2,255 and 4,048 central line days, 2,124 and 3,888 ventilator days, and 3,708 and 5,106 urinary catheter days, respectively, were reported. The utilization ratio increased by 10.7% for central lines and 12.9% for ventilators, while a reduction of 15% in the urinary catheter utilization ratio was observed. DAI incidence was higher during the COVID-19 pandemic, with a significant increase for VAP [from 0.47 to 7.2 per 1,000 ventilator days], CLABSI [from 1.77 to 4.94 per 1,000 central line days], and CAUTI [from 0.54 to 1.76 per 1,000 urinary catheter days]. This represents an increase in the risk of infection by 2.79 (95% CI: 0.93–11.21; *p* < 0.05), 15.31 (2.53–625.48; *p* < 0.00), and 3.25 (0.68–31.08; *p* < 0.012) for CLABSI, VAP, and CAUTI, respectively (*p* < 0.00) (Table 4). Table 5 shows the DAI rates according to the published reports.^6,10,18–21^

## Discussion

This is the first report that provides data useful for benchmarking the incidence of HAI and the impact of coronavirus infection in a single facility in Qatar. It highlights the increased risk of infection related to the COVID-19 pandemic, with special reference to VAP and CLABSI.

The incidence of DAI during the study period shows lower results than those reported by the International Nosocomial Infection Control Consortium (2013–2018) and the Kingdom of Saudi Arabia (2013–2016).^19,22^ The incidence of CLABSI and CAUTI was lower than that reported by the National Healthcare Safety Network (NHSN, USA) (2012), while for VAP, it was higher than that reported by the NHSN (2013).^20,21^ The effect of the pandemic period that contributed to the increase in the pooled mean for the 10-year data should be considered for setting the facility’s infection prevention program goals.

The ICU changed substantially in complexity during the pandemic in comparison with the pre-pandemic period, mainly related to patients’ population characteristics. From a pre-pandemic population with low HAI risks to COVID-19 patients with high HAI risks. This is determined by the COVID-19 cases’ clinical severity, treatment with antibiotics and steroids, and the need for invasive devices, especially mechanical ventilation, and central venous, and arterial lines. These factors play a paramount role in explaining the increased incidence of HAI during the pandemic, in addition to compliance with infection control practices.^3,23,24^

It is worth noting the frequency of other respiratory infections, not classified as VAP, that may correspond to tracheobronchitis in ventilated patients.^25,26^ A multicenter study conducted in 36 European ICUs showed an increased incidence of ventilator-associated lower RTIs in COVID-19 patients, and 28% of these infections were related to tracheobronchitis.^25^

Although few cases have been reported in the COVID-19 pandemic period, confirmed patients with BSIs related to peripheral lines should be highlighted. Previous references to the risk of infection associated with these devices required prevention practices like those recommended for central lines.^27,28^

The increase in CLABSI and VAP incidence and the non-increase in CAUTI incidence during the COVID-19 pandemic have been reported previously.^3,12^ Concerning the risk of CAUTI, it is worth considering that although urinary catheters were intensively used in patients with coronavirus, the utilization ratio was lower than that observed during the pre-pandemic period.

The etiology of the reported infections was similar to that previously described, except for the high frequency of infections caused by Candida species, especially cases of CLABSI and *Candida auris*. Most of the patients with *Candida auris* infections had previously reported colonization.^29–31^

An increase in antimicrobial resistance has been reported as a principal consequence of the pandemic,^29,31^ including bacterial and fungal resistance, but the number of isolations during the study should not be considered conclusive data to confirm this finding. However, the increase in the frequency of multidrug-resistant species like *Enterobacter*, *Klebsiella*, *Pseudomonas*, *Serratia marcescens*, and *Stenotrophomonas maltophilia* should point to the need to strengthen our policies and practices to contain the emergency of antimicrobial resistance.

The study has a few limitations, including a retrospective observational design being single-center, which limits data comparison to ICUs with similar characteristics. The scope of analysis should also consider the few multidrug-resistant isolated organisms. The impact of data collection by various infection control staff (mainly for the period 2017–2019) was minimized by accurate data validation. The study’s main strength is the use of standardized surveillance systems and procedures guided by a corporate infection control program.

## Conclusion

The incidence of DAI increased during the pandemic period as compared to the pre-pandemic period, and limited evidence of the impact on antimicrobial resistance was observed. The infection control program should evaluate strategies to minimize the impact of pandemics on HAI.

### Acknowledgment

We thank Prof. Alexis Gonzalez Velázquez for proofreading the manuscript.

### Ethical statement

The study was approved by The Medical Research Center (Hamad Medical Corporation, Doha, Qatar) with MRC-01-22-223.

### Authors’ contributions

Study design: HGG. Data acquisition: HGG, AVA. Data analysis: HGG, JAA, AVA, JCA, AMFG, TMFH. Manuscript writing: HGG. Critical review and major scientific input: HGG, JAA, AVA, JCA, AMFG, TMFH.

## Figures and Tables

**Table 1.** HAIs in the pre-COVID-19 (2012–2019) and COVID-19 (2020–2021) periods (The Cuban Hospital Medical Surgical Intensive Care Unit).

**Infections**	**Pre-COVID-19** **(*n* = 22)**	**COVID-19** **(*n* = 130)**
DAI		
CAUTI	2 (9.1%)	13 (10.0%)
CLABSI	1 (4.5%)	30 (23.1%)
VAP	1 (4.5%)	30 (23.1%)
BSI related to the peripheral venous line	–	2 (1.5%)
Others – HAI		
Others – respiratory infections	17 (77.3%)	46 (35.4%)
*Clostridium difficile* infections	1 (4.5%)	–
DECU	–	4 (3.1%)
Skin infections	–	5 (3.8%)
SUTI 1b	–	1 (0.8%)

BSI: bloodstream infection; CAUTI: catheter-associated urinary tract infection; CLABSI: central-line bloodstream infection; DECU: decubitus ulcer infection; HAI: healthcare-associated infections; SUTI: symptomatic urinary tract infection; VAP: ventilator-associated pneumonia.

**Table 2.** Etiology of HAI in the pre-COVID-19 (2012–2019) and COVID-19 (2020–2021) periods (The Cuban Hospital Medical Surgical Intensive Care Unit).

**Isolate**	**Pre-COVID-19**		**COVID-19**	
	**Total no. isolates**	**MDRO (%)** ^*^	**Total no. isolates**	**MDRO (%)** ^*^
Bacterial				
*Acinetobacter* spp.	0		6	0.0
*Citrobacter* spp.	0		2	0.0
*Enterobacter* spp.	3	33.3	12	91.7
*Enterocococcus* spp.	0		9	11.1
*Escherichia coli*	0		6	83.3
*Klebsiella* spp.	6	16.7	30	46.7
*Morganella morgani*	1	100	1	100.0
*Proteus* spp.	2	50.0	0	
*Pseudomonas* spp.	5	20.0	42	40.5
*Serratia marcescens*	2	50	11	100.0
*Staphylococcus aureus*	2	50	3	66.6
*Stenotrophomonas maltophilia*	1	100	9	66.7
*Clostridium difficile*	1	100	0	0
Fungal^**^				
*Candida* spp.	0		13	
*Trichosporon asahii*	1		0	

^*^Proportion of isolated with a multidrug-resistant pattern, ^**^resistance to antifungal not presented, #spp. refers to species. HAI: healthcare-associated infections; MDRO: multidrug-resistant organisms.

**Table 3.** Pooled means and key percentiles of the distribution of DAI rates and device utilization ratios (The Cuban Hospital Medical Surgical Intensive Care Unit 2012–2021).

**Infection**	**Infections**	**Device days**	**Pooled mean**	**10%**	**25%**	**50% (median)**	**75%**	**90%**
CAUTI	15	8,814	0.8	0.0	0.0	0.0	2.2	2.6
CLABSI	31	6,303	2.6	0.0	0.0	0.0	7.1	8.4
VAP	31	6,012	2.9	0.0	0.0	0.0	6.3	13.5
Devices	Patients’ days	Device days	Pooled mean	10%	25%	50% (median)	75%	90%
Urinary catheter	19,204	8,814	0.49	0.37	0.42	0.46	0.55	0.66
Central line	19,204	6,303	0.32	0.25	0.30	0.33	0.34	0.35
Ventilator	19,204	6,012	0.31	0.20	0.21	0.30	0.39	0.49

CAUTI: catheter-associated urinary tract infection; CLABSI: central-line bloodstream infection; DAI: device-associated infection; VAP: ventilator-associated pneumonia.

**Table 4.** Device utilization ratio and infection rate (per 1000 device days) during 2012–2019 (pre-COVID period) and 2020–2021 (COVID-19 period).

**HAI type**	**Pre-COVID-19 (2012–2019)**			**COVID-19 (2020–2021)**			**RR (95% CI)**
	**Device days, *n***	**Infections, *n***	**HAI rate**	**Device days, *n***	**Infections, *n***	**HAI rate**	
CAUTI	3,708	2	0.54	5,106	9	1.76	3.25 (0.68–31.08)^***^
CLABSI	2,255	4	1.77	4,048	20	4.94	2.79 (0.93–11.21)^*^
VAP	2,124	1	0.47	3,888	28	7.20	15.31 (2.53–625.48)^**^

^*^p < 0.05, ^**^p = 0.00, ^***^p = 0.12. HAI: healthcare-associated infections; RR: relative risk; CAUTI, catheter-associated urinary tract infection; CLABSI, central-line bloodstream infection; VAP, ventilator-associated pneumonia.

**Table 5.** Summary data of device utilization ratio and infection rate (per 1,000 device days) in various reports.

**Reports** ^*^	**Population**	**CLABSI**		**VAP**		**CAUTI**	
		**Infection rate**	**Utilization ratio**	**Infection rate**	**Utilization ratio**	**Infection rate**	**Utilization ratio**
NHSN 2012	Medical surgical ICU ≤ 15 beds			1.1	0.24		
	Medical surgical ICU > 15 beds			0.9	0.34		
NHSN 2013	Medical surgical ICU ≤ 15 beds	0.8	0.37			1.3	0.54
	Medical surgical ICU > 15 beds	0.8	0.49			1.7	0.63
INICC 2013–2018	Medical surgical ICUs	4.4	0.53	11.13	0.38	2.97	0.67
KSA 2013–2016	Medical surgical ICU ≤ 15 beds	0–6.9	0.58	18.1–26.6	0.54	2.3–7.19	0.75
	Medical surgical ICU > 15 beds	0–22.8	0.33–0.68	0.9–186	0.05–0.94	0–11.75	0.06–0.85
US hospitals 2019–2020	Critical care units 78 US hospital pre-COVID-19 period	0.68	0.43			0.88	0.48
	Critical care units 78 US hospital COVID-19 period	1.16	0.48			0.90	0.51
INICC 2022	Medical surgical ICUs pre-COVID-19 period	2.54		9.71		1.64	
	Medical surgical ICUs COVID-19	4.73		12.58		1.43	

^*^References 6, 10, 16–19. ICU: intensive care unit; INICC: International Nosocomial Infection Control Consortium; NHSN: National Healthcare Safety Network; CAUTI, catheter-associated urinary tract infection; CLABSI, central-line bloodstream infection; VAP, ventilator-associated pneumonia.
